# Bioinformatics in Jordan: Status, challenges, and future directions

**DOI:** 10.1371/journal.pcbi.1007202

**Published:** 2019-09-12

**Authors:** Qanita Bani Baker, Maryam S. Nuser

**Affiliations:** 1 Department of Computer Science, Jordan University of Science and Technology, Irbid, Jordan; 2 Department of Information Systems, Yarmouk University, Irbid, Jordan; CPERI, GREECE

## Abstract

Bioinformatics plays a key role in supporting the life sciences. In this work, we examine bioinformatics in Jordan, beginning with the current status of bioinformatics education and research, then exploring the challenges of advancing bioinformatics, and finally looking to the future for how Jordanian bioinformatics research may develop.

## Author summary

Bioinformatics is an important multidisciplinary field for many life sciences. Mathematics, statistics, computer science, and the life sciences join together to breathe life into the discipline of bioinformatics. In Jordan, rapid progress in life science, research, and healthcare have created a demand for the bioinformatics field. Because of the importance of bioinformatics and the limited attention to it in Jordan, a need arises to convince stakeholders about supporting this field in the country. We discuss the current status of bioinformatics efforts in education and research in Jordan and investigate the challenges affecting the development of bioinformatics in the country. We present future directions and the opportunities for further development of the discipline. This work is a core step towards a capacity-building plan to attract bioinformatics experts to support bioinformatics research and education in the near term in Jordan. We believe that the current challenges of limited educational opportunities, relatively little attention from Jordanian funding agencies for bioinformatics, and few conferences that bring visitors who are leaders in bioinformatics into Jordan demand focused attention to remedy.

## Introduction

Previously published studies on the development of bioinformatics in several countries [1–7] inspired us to investigate the status of bioinformatics in Jordan. Bioinformatics is a vast multidisciplinary field that has developed computational tools to analyze and manage constantly growing amounts of biological data [8]. The increasing importance of integrative approaches to understanding biology and a burgeoning interest in personalized medicine make bioinformatics essential in many life science projects around the world [9]. In Jordan, rapid progress in life science research and healthcare has created a demand for bioinformatics [10, 11], especially with a rapidly growing set of biological data repositories [12].

In this work, we investigate the current status of bioinformatics education and research in Jordan, summarize the number of Jordanian bioinformatics publications and compare this with those of other Arab countries, analyze the proportion of bioinformatics reports within the Jordanian biomedical literature, and discuss future directions and opportunities for expansion of bioinformatics in Jordan.

## Current status

### Centers of bioinformatics research and education in Jordan

There are no devoted bioinformatics institutes in Jordan, but there are at least four centers that feature bioinformatics as an important part of their research and education portfolios.

The Princess Haya Biotechnology Center (PHBC) was established in 2013 at the Jordan University of Science and Technology (JUST) to support research in biotechnology at the national and regional levels. The PHBC envisions leading genomics, proteomics, metabolomics, and bioinformatics in Jordan. The PHBC provides training in all these areas, including bioinformatics.

The Synchrotron-light for Experimental Science and Applications in the Middle East (SESAME) is another center supporting bioinformatics research and education in Jordan. SESAME is not a bioinformatics-specialized center, but with its research portfolio that includes biology, archaeology, and the medical sciences, bioinformatics plays a fundamental role that is recognized and supported at SESAME.

The Jordanian Society for Microbial Biodiversity (JSMB) focuses on the study and conservation of microbial biodiversity. Like PHBC and SESAME, JSMB makes extensive use of bioinformatics and has a bioinformatics research program to support investigations of microbial diversity. Finally, the International Academy of Pathology–Arab Division (IAP–AD) is a center for research that also recognizes the important supporting role of bioinformatics in pathology.

### Jordanian conferences and workshops in bioinformatics

Jordan has hosted several conferences and workshops that were either devoted to or have featured bioinformatics. For example, the Department of Biotechnology and Genetic Engineering of Philadelphia University (Jordan), in collaboration with the Institute of Genetic and Genomic Studies at the University of Tübingen (Germany), organized the First German-Jordanian Meeting: Molecular Genetics of Human Diseases in 2015. The focus of the conference was on modern techniques in molecular biology and the diagnosis of genetic diseases, particularly those that cause nondevelopmental mental retardation in some families in Jordan. Given this theme, presentations on bioinformatics tools and approaches were an important part of this conference.

Bioinformatics also was featured heavily in the International Conference on Ethics & Biomedical Informatics organized by the College of Pharmacy at JUST in collaboration with the University of Miami Bioethics Program and Responsible Conduct of Research (RCR) Program.

The first International Conference on Cancer Care Informatics, held in 2018 in Amman, Jordan, also prominently featured bioinformatics, including the workshop Bioinformatics for Cancer Studies. The conference was organized by King Hussein Cancer Foundation and, in partnership with the University of the West of England, Bristol, UK, featured six keynote speakers that included bioinformatics experts.

### Degrees

Currently, there are no dedicated bioinformatics degree programs in Jordan. Instead, largely isolated courses in bioinformatics are offered within computer science and information technology departments. Table 1 illustrates the course name, the department they are offered under, and the university name. In many cases, instructors who teach these courses are not practicing bioinformaticians. To advance the field and to train scientists in bioinformatics in Jordan, a dedicated bioinformatics degree program with faculty who are specialists in bioinformatics must be established.

**Table 1:** The distribution of bioinformatics courses within different departments in Jordanian universities.

Course Name	Department	University	Course Content Summary
Bioinformatics	Department of Horticulture and Crop Science	UJ	Basic molecular biology. Biological diversity. Sequence manipulation: gene identification, homology searching, alignment, PSI-BLAST. Phylogenetic analysis. Protein structure: acquisition, prediction, analysis of function.
Biocomputing	Department of Biotechnology and Genetic Engineering	JUST	Introduction to biotechnology databases such as GenBank/DDBJ/EMBL, OMIM, PubMed, PDB, Enzymes, and SwissProt. Analyze DNA, RNA, and protein sequences. Similarity search. Predict genes and protein secondary structures multiple sequence alignments and phylogenetic trees. Lasergene software package.
Fundamentals of Bioinformatics	Computer Science	JUST	Overview of bioinformatics with a significant problem-solving component, including hands-on practice using computational tools to solve a variety of biological problems. Topics include database searching, sequence alignment, gene prediction, RNA and protein structure prediction, comparative and functional genomics, and construction of phylogenetic trees.
Advanced Bioinformatics	Computer Science	JUST	Introduction to bioinformatics, bioimage informatics, multiscale modeling, sequence alignment and multiple alignments, motif finding, big data analytics in bioinformatics, high-performance computing for bioinformatics, and machine learning in bioinformatics.
Bioinformatics	Biomedical Engineering	JUST	An interdisciplinary effort between molecular biology and computer science aimed at extracting the relevant biological information from the genome and overview of data mining, data analysis, and computational methods of DNA, RNA, and proteins, as well as major applications and research areas.
Bioinformatics I	Biomedical Systems and Informatics Engineering	YU	Introduction to bioinformatics and organizing and maintaining a large volume of genomic data. Genome sequencing projects, proteomics, and gene-expression studies. Principles and simulation methodologies for the integration of genomic and physiological data in the analysis of complex biological processes and for diagnostic matters.
Bioinformatics Lab	Biomedical Systems and Informatics Engineering	YU	This is a lab that emphasizes the hands-on application of bioinformatics methods to biological problems: sequence alignment, fast database search, profiles and motifs, comparative genomics, gene finding, phylogenetic trees, protein structure, functional characterization of proteins, expression analysis, and computational proteomics.
Bioinformatics II	Biomedical Systems and Informatics Engineering	YU	Apply fundamental bioinformatics methods to analyze protein sequence and structure data, genomic DNA sequence, and gene-expression data. Interpret and evaluate results of key bioinformatics analyses; system biology focusing on the development of models on molecular and tissue level. Design effective strategies for application of bioinformatics methods and combine fundamental methods into multipart strategies for addressing complex problems.
Computational Biology	Biomedical Systems and Informatics Engineering	YU	Modeling and analysis of biological systems. The multiscale modeling of biological systems, modeling strategies. Compartmental models of physiologic systems. Cellular models, organ models, systems models. Methods and tools for identification. Analysis of molecular biology databases, sequence analysis, modeling of regulatory networks and metabolic pathways.
Introduction to bioinformatics	Computer Science	GJU	Introduction to bioinformatics: principles, concepts, methods, and strategies to transform and process the masses of information from biological experiments; DNA and protein sequence alignment and analysis; database searching; RNA folding.
Bioinformatics	Biotechnology and Genetic Engineering	PU	Biological databases, sequence alignment, molecular phylogeny, and human genome.

**Abbreviations:** DDBJ, DNA Data Bank of Japan; EMBL, European Molecular Biology Laboratory; GJU, German Jordanian University; JUST, Jordan University of Science and Technology; OMIM, Online Mendelian Inheritance in Man; PDB, Protein Data Bank; PSI-BLAST, Position-Specific Iterative Basic Local Alignment Search Tool; PU, Philadelphia University; UJ, The University of Jordan; YU, Yarmouk University.

### Jordanian publications in bioinformatics

**Fig 1:**
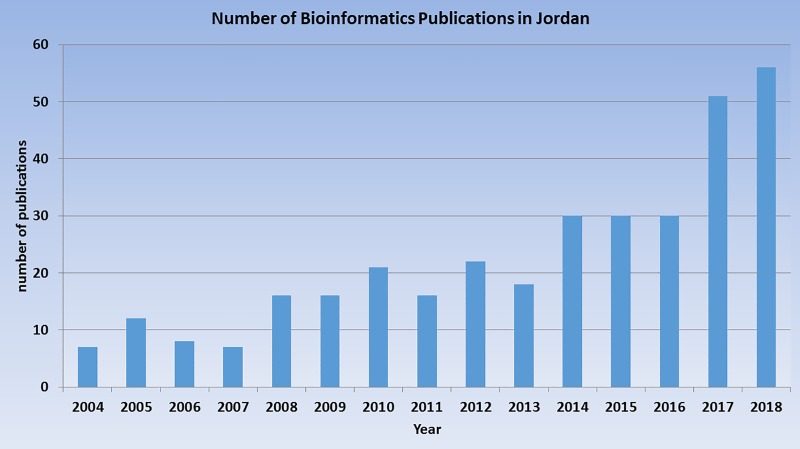
Bioinformatics-related publications authored by scientists affiliated with Jordanian institutions. The results show the number of hits obtained in a search of the Scopus database using the terms “next-generation sequencing,” “computational biology,” “bioinformatics,” “genomic,” and “in silico”.

We used the query terms (“next-generation sequencing,” “computational biology,” “bioinformatics,” genomic,” and “in silico”) used by the authors in [7] to search the Scopus [13] and PubMed [14] databases for the period from 2004–2018 and filtered these hits to include only authors affiliated with Jordanian institutions. As seen in Fig 1, there was a sharp rise in the number of publications from Jordanian universities and research centers with keywords that matched these search terms that identify publications relevant to bioinformatics.

**Fig 2:**
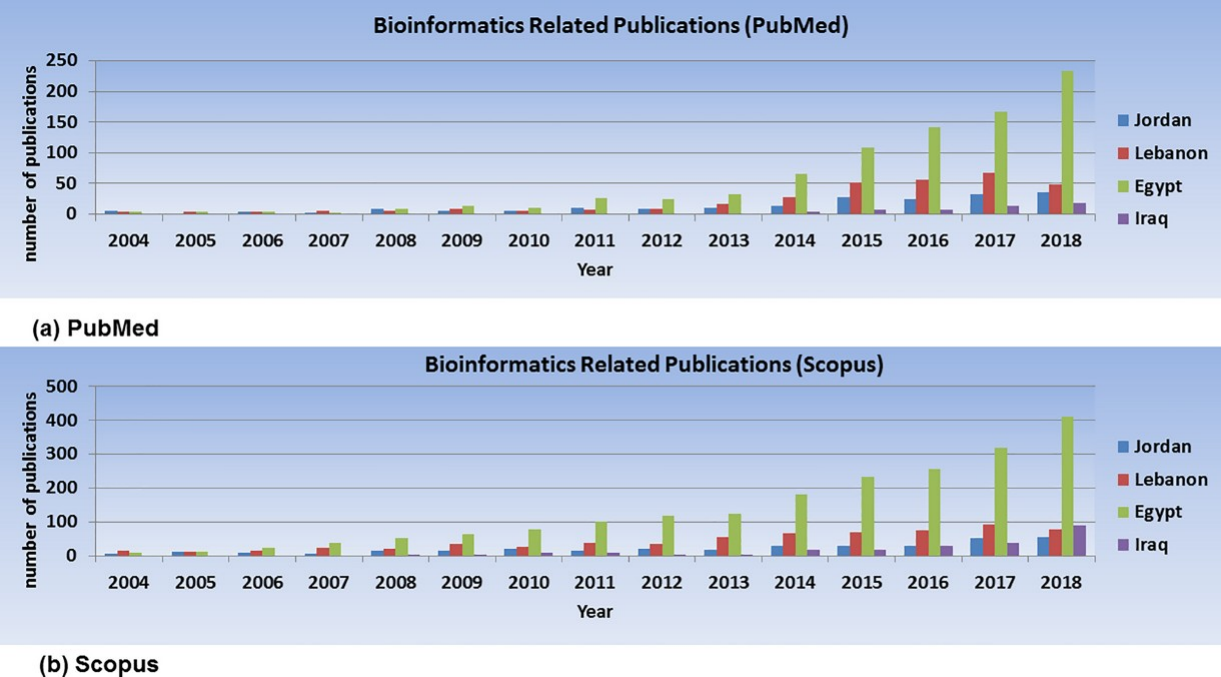
Bioinformatics-related publications authored by scientists affiliated with Jordanian institutions compared to publications authored by scientists affiliated with Lebanon, Egypt, and Iraq. The PubMed (a) and the Scopus (b) databases were searched, and the hits were sorted into categories of publications from scientists affiliated with these four Arab countries.

The number of bioinformatics-related publications authored by scientists affiliated with Jordanian institutions was compared to the number of publications authored by scientists from other Middle Eastern countries with an economy relatively close to Jordan’s based on World Bank (http://www.worldbank.org/) classification [15]. These countries are Lebanon, Egypt, and Iraq. As seen in Fig 2, Jordan lags behind Egypt and Lebanon in the number and growth of publications in bioinformatics.

**Fig 3:**
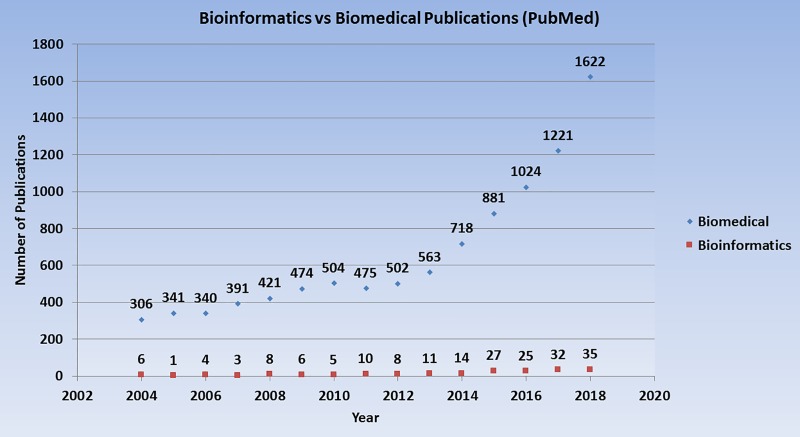
Bioinformatics-related publications compared with biomedical-related publications authored by scientists affiliated with Jordanian institutions between 2004 and 2018.

We also used the PubMed database to compare the growth in Jordanian bioinformatics publications relative to the growth in publications in the broadly defined field of biomedical sciences. These results, which are shown in Fig 3, indicate that the growth of Jordanian bioinformatics publications lags far behind the growth in biomedical publications.

## Challenges to the growth of bioinformatics in Jordan

In this section, we discuss challenges to the development of bioinformatics in Jordan:

1. There is a lack of scientists in Jordan with expertise in bioinformatics. The absence of even a single dedicated degree program in bioinformatics is at least partially to blame. For whatever reason, the need to strengthen bioinformatics to support the expansion of the life sciences in Jordan has gotten little attention.

2. Short-term, targeted educational programs, such as workshops and train-the-trainer courses, are rare in Jordan. These programs have the potential to spark interest in bioinformatics in Jordanian researchers and students. More educational programs must be instituted if bioinformatics is to flourish in Jordan.

3. Much of the needed infrastructure for bioinformatics such as high-performance computers, operating and information systems, and software is lacking in Jordan. Expanding this computing infrastructure would go a long way to increasing the development and application of bioinformatics in Jordan [16].

4. The funding needed to develop robust bioinformatics research and education programs is difficult to obtain in Jordan. Funding needs to be expanded to have a chance of developing a robust bioinformatics program in the country.

## Future directions

As outlined above, there are significant challenges to expanding the scope and quality of bioinformatics research in education in Jordan. Here, we offer some suggestions for meeting these challenges.

1. **Short-term training, workshops, and conferences:** Opening more opportunities for training in bioinformatics may be one of the easiest steps to improving the state of bioinformatics in Jordan. Short training courses delivered face to face or online should be offered more frequently for current bioinformatics researchers and for those interested in learning about the field. To design such short courses, we suggested taking the benefits from the case studies shown in [12, 17, 18]. In order to find sustainable solutions, we suggest finding train-the-trainer courses that aim to keep lecturers and researchers updated in this dynamic field will also be important. Such courses can be set up in collaboration with international bioinformatics institutes. Similarly, increasing the number of conferences that focus solely on bioinformatics or feature it as an important component will provide valuable opportunities for education and peer-to-peer networking among Jordanian scientists, as well as increasing international collaborations. These steps can support the competencies needed for the number of researchers working in several fields related to bioinformatics in the country. As reported by The Higher Council for Science and Technology (HCST), Jordan currently has around 10,519 PhD researchers working in several research fields, with 1,003 in Information Technology, 282 in Biology, 339 in Mathematics and Statistics, 656 in Physics, and 1,307 in the medical fields.

2. **Creation of bioinformatics degree programs:** The lack of bioinformatics degree programs in Jordan is an obvious impediment to expanding the field. Comprehensive bionformatics degree programs at the undergraduate and graduate levels must be created to train Jordanian scientists. As a start, BS-, MS-, and PhD-degree–granting programs patterned after respected programs at United States and European universities need to be established in at least one of Jordan's major centers of learning.

3. **Develop and enhance the courses curricula:** Introducing bioinformatics courses into computer science and life sciences curricula would help develop multidisciplinary scientists who are proficient in using bioinformatics and who will be able to advance bioinformatics. Funding to provide doctoral and postdoctoral research fellowships in bioinformatics is another critical element to move Jordanian bioinformatics forward. These fellowships should be part of the strategic plan for the Jordanian Higher Education Ministry.

4. **Investment in computing power and data storage:** Bioinformatics development benefited from open sources that are publicly available [19]. Several of these tools are cloud-based tools, with most of the computations being processed in the cloud platforms [20]. Since there is poor information and communications technology infrastructure in some areas in the country, educational and research institutes can invest in Free and Open-Source Software (FOSS) resources to improve bioinformatics knowledge and enhance the education platforms. For data-intensive applications in terms of processing power and storage space, the supercomputer infrastructures, such as the IMAN1 project, can be utilized.

5. **Foster national and international collaborations:** Programs that encourage collaborations between bioinformatics researchers within Jordan and between Jordanian researchers and international colleagues will spur the development of bioinformatics in the kingdom. We suggest funding a national bioinformatics network that is similar to the Asia Pacific Bioinformatics Network (APBioNet). We also recommend an increased role for SESAME as a center for bioinformatics research and as a host for conferences and workshops to bring together Jordanian bioinformaticists and international bioinformatics researchers. Finally, Jordanian scientists should be encouraged to engage with international organizations that promote bioinformatics, such as the International Society for Computational Biology (ISCB), the Global Organization for Bioinformatics Learning, Education & Training (GOBLET), and Bioinformatics Club for Experimenting Scientists (Bioclues).

6. **Increased funding for bioinformatics:** Of course, increases in funding are demanded by scientists in every discipline. But with a documented paucity of Jordanian bioinformatics publications relative to those of other Arab nations, coupled with the essential supporting role that bioinformatics plays in so many fields, the case for increased funding for bioinformatics is compelling.

## Conclusion

There are significant challenges and opportunities in furthering the development of bioinformatics in Jordan. The good news is that the current challenges of limited educational opportunities, relatively little attention from Jordanian funding agencies for bioinformatics, and few conferences that bring visitors who are leaders in bioinformatics into Jordan are all relatively easy to remedy. The strong support for biomedical research, coupled with the essential role of bioinformatics in many areas of biomedicine, bodes well for strengthening bioinformatics research and education in the near term in Jordan.
